# Lessons Learned and Paths Forward for Rabies Dog Vaccination in Madagascar: A Case Study of Pilot Vaccination Campaigns in Moramanga District

**DOI:** 10.3390/tropicalmed6020048

**Published:** 2021-04-12

**Authors:** Caitlynn Filla, Malavika Rajeev, Zoavina Randriana, Chantal Hanitriniana, Radoniaina R. Rafaliarison, Glenn Torrencelli Edosoa, Mamitiana Andriamananjara, Nivohanitra P. Razafindraibe, José Nely, Angelique Ferreira, Annie L. Yang, Fenomanana Daniel, Tara A. Clarke, Zachary Farris, Terry Stone, Jochem Lastdrager, Tsiky Rajaonarivelo, Katie Hampson, C. Jessica E. Metcalf, Kim Valenta

**Affiliations:** 1 Department of Anthropology, University of Florida, Gainesville, FL 32611, USA; cfilla@ufl.edu (C.F.); kimvalenta@ufl.edu (K.V.); 2 Department of Ecology and Evolutionary Biology, Princeton University, Princeton, NJ 08544, USA; mrajeev08@gmail.com (A.L.Y.); cmetcalf@Princeton.edu (C.J.E.M.); 3 The Mad Dog Initiative Akanin’ny Veterinera, Akaikiniarivo, Ambatobe, Antananarivo 101, Madagascar; rzoavina@gmail.com (Z.R.); radorafaliarison@gmail.com (R.R.R.); angique@gmail.com (A.F.); fenomanana17@gmail.com (F.D.); taclarke@ncsu.edu (T.A.C.); farriszj@appstate.edu (Z.F.); th.rajaonarivelo@gmail.com (T.R.); 4 Mention Zoologie et Biodiversité Animale, Faculté des Sciences, Université d’Antananarivo, Antananarivo 101, Madagascar; hanitriniainachantal@gmail.com; 5 Chargé des Maladies Tropicales Négligées Organisation Mondiale de la Santé Madagascar, Antananarivo 101, Madagascar; edosoag@who.int; 6 Direction des Services Vétérinaires Ministère Chargé de l’Agriculture et de l’Élevage, Antananarivo 101, Madagascar; andriamana_njara@ymail.com (M.A.); nhperle@gmail.com (N.P.R.); 7 Service contre les Maladies Endémo-épidémiques et Tropicales Négligées Ministère de la Santé Publique, Antananarivo 101, Madagascar; josenely@yahoo.fr; 8 Travelling Animal Doctors, Newark, DE 19711-2916, USA; TerryWStone9@gmail.com (T.S.); J.J.Lastdrager@gmail.com (J.L.); 9 Department of Sociology and Anthropology, North Carolina State University, Raleigh, NC 27695-8107, USA; 10 Department of Health and Exercise Science, Appalachian State University, Boone, NC 28608, USA; 11 Boyd Orr Centre for Population and Ecosystem Health Institute of Biodiversity, Animal Health and Comparative Medicine University of Glasgow, Glasgow G12 8QQ, UK; Katie.Hampson@glasgow.ac.uk

**Keywords:** canine rabies, mass dog vaccination, central point vaccination, puppy vaccination, Zeroby30

## Abstract

Canine rabies causes an estimated 60,000 human deaths per year, but these deaths are preventable through post-exposure prophylaxis of people and vaccination of domestic dogs. Dog vaccination campaigns targeting 70% of the population are effective at interrupting transmission. Here, we report on lessons learned during pilot dog vaccination campaigns in the Moramanga District of Madagascar. We compare two different vaccination strategies: a volunteer-driven effort to vaccinate dogs in two communes using static point vaccination and continuous vaccination as part of routine veterinary services. We used dog age data from the campaigns to estimate key demographic parameters and to simulate different vaccination strategies. Overall, we found that dog vaccination was feasible and that most dogs were accessible to vaccination. The static-point campaign achieved higher coverage but required more resources and had a limited geographic scope compared to the continuous delivery campaign. Our modeling results suggest that targeting puppies through community-based vaccination efforts could improve coverage. We found that mass dog vaccination is feasible and can achieve high coverage in Madagascar; however, context-specific strategies and an investment in dog vaccination as a public good will be required to move the country towards elimination.

## 1. Introduction

Canine rabies results in an estimated 60,000 human deaths per year globally [1]. These deaths are entirely preventable: prompt post-exposure prophylaxis of humans exposed to rabies is highly effective at preventing death and mass dog vaccination can interrupt transmission in domestic dogs and eventually lead to disease elimination [2]. The World Health Organization (WHO) and its partners have set a goal to eliminate human deaths due to canine rabies by the year 2030 (“ZeroBy30”) [3]. Annual dog vaccination campaigns achieving at least 70% coverage are the recommended target for controlling rabies in domestic dog populations [4]. However, achieving this coverage target in low- and middle-income countries where the burden of human rabies is highest can be challenging due to economic, ecological, sociocultural, and political barriers [5].

In sub-Saharan African countries, parenteral vaccinations implemented through static point campaigns have been shown to be cost-effective and feasible [6]. While most dogs in these settings are considered free roaming, they are mostly owned and are accessible for vaccination through these campaigns [7,8]. However, reaching high coverage and maintaining vaccination campaigns at that scale requires sustained investment and coordination, and the challenges in implementation largely reflect financial and logistical constraints more than the feasibility of vaccination itself [5]. Developing clear and context-specific strategies and lowering costs and resources needed could help spur the implementation and scaling up of campaigns in these countries.

In Madagascar, canine rabies has been an endemic for over a century, and for most of that period, the Institut Pasteur de Madagascar has provided post-exposure prophylaxis free-of-charge to bite patients in the country [9]. Currently, there are only 31 clinics where these human vaccines are available, with one clinic serving on average greater than 700,000 persons and over 20,000 bite patients treated annually [10]. There is minimal dog vaccination due to high costs to owners and a lack of vaccine availability [11]. Recent studies have estimated a high burden of human rabies deaths (approximately 1000 deaths annually), masked by weak surveillance across the country [10,11].

The veterinary sector is largely private and practices are largely limited to urban areas, but approximately 204 veterinarians are employed in hybrid private/public employment as designated district veterinarians by the national government. While dog vaccination is rare, livestock officers and veterinarians work together to implement cattle vaccination campaigns for anthrax on an annual basis as mandated by the government (owners are charged a fee per animal for these vaccines, which vary by location) [12]. No routine mass dog vaccinations have been conducted on the island, although a few pilot programs have begun in recent years, largely implemented by NGO–government partnerships.

Here, we summarize lessons learned through the implementation of pilot vaccination programs in the Moramanga District of Madagascar, where previous work has shown high incidence of dog rabies cases and human rabies exposures. In 2018 and 2019, we deployed two different vaccination strategies. In 2018, we carried out a larger scale volunteer-led pilot vaccination campaign in two communes (sub-district level) using a static point strategy where owners brought their animals to a fixed location for vaccination. In 2019/2020, we provided vaccines, all necessary supplies for vaccine administration, and a per-vaccine fee to the district veterinarian to vaccinate animals as part of a continuous vaccination strategy, where vaccines were delivered alongside routine services provided by the veterinarian. We compare the time, human resources, costs, and coverage estimates between these campaigns, and using a demographic and vaccination model, we further explore different vaccination strategies based on what we learned during implementation.

## 2. Methods

### 2.1. Study Area

The Moramanga District is located midway between the central highlands and the east coast of Madagascar at an average altitude of 936 m. It comprises 21 communes, covering approximately 7150 km^2^ with an approximate human population of 347,000 [13]. Previous work in the district has established a high burden of rabies exposures (42–110 per 100,000 persons) and deaths (1–3 deaths per 100,000 persons) despite the availability of post-exposure prophylaxis at the district hospital [11]. While Moramanga is relatively close to the capital city of Antananarivo (~3 h by bus), within the district, travel times between locations are highly variable, with much of the population living in more rural areas with limited access to roads and transportation [10]. Before 2018, there were limited animal rabies vaccination services, with most animal vaccines available in the urban commune of Moramanga Ville, where owners were often charged > 15,000 Ariary (~4.28 USD) per vaccine administered.

### 2.2. 2018 Campaign

**Figure 1:**
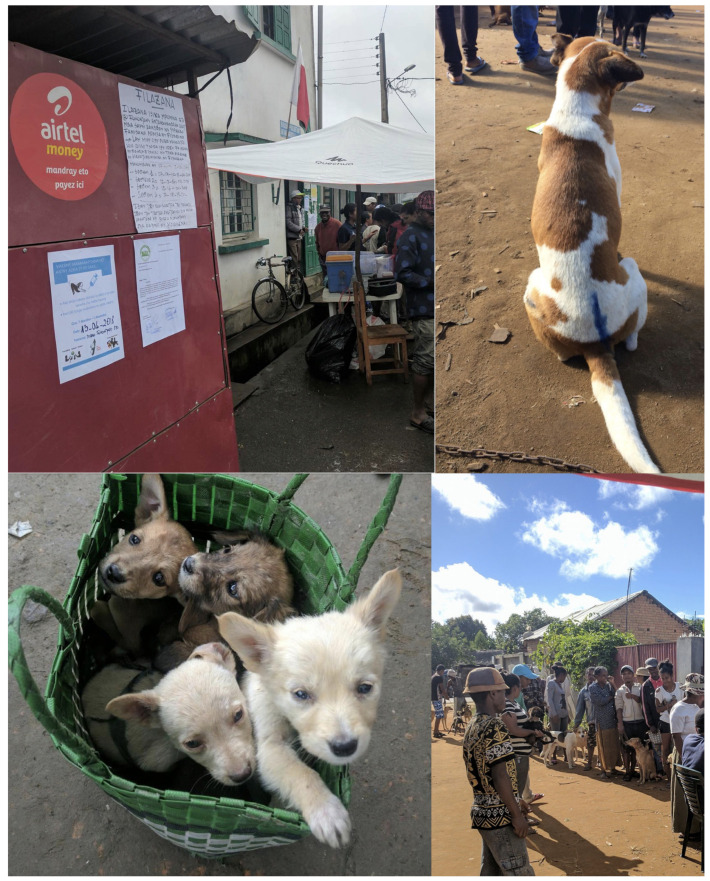
Photos from the 2018 campaign. **Top left**: advertisement for the campaign posted on the door of the fokontany office as the campaign starts; **top right**: a dog post-vaccination marked with a crayon; **bottom left**: a basket of puppies brought for vaccination; and **bottom right**: a line of owners and dogs waiting for vaccination. Photo credit: Jochem Lastdrager, Traveling Animal Doctors.

In 2018, two NGOs (the Mad Dog Initiative (MDI) and Traveling Animal Doctors (TAD)) organized a pilot vaccination campaign in collaboration with the Department of Veterinary Services and the Ministry of Public Health in the District of Moramanga Figure 1. This campaign focused on two communes in Moramanga, Moramanga Ville (the district center) and Andasibe (a rural commune surrounding Andasibe National Park), where previously high incidence of probable rabies exposures (Moramanga Ville) and a high burden of deaths (Andasibe) had been recorded [11].

The campaign was planned as a series of static point vaccination stations covering 1–3 fokontany (i.e., sub-communes) per day (see Figure 1). A week before the campaign dates, the vaccination team informed the chief of the fokontany about the campaign and provided fliers advertising the date of the vaccine and that it would be available at no cost to owners (Figure 1). During the campaign, we used Rabisin (10 mL vials with 1 mL per dose, Boehringer Ingelheim) to vaccinate both dogs and cats presented that were at least 1 month old based on current WHO recommendations for endemic settings [4,14]. Rabisin has a manufacturer-stated duration of protection of 1 year given one dose and of an additional three years if an additional dose is given approximately one year after the first dose. As part of the campaign, owners were surveyed by vaccinators about how many dogs and cats they owned in total (split by >1 year vs. <1 year in order to avoid language ambiguities that might result in excluding puppies and kittens) as well as if their dogs were free roaming (no restrictions on movement by the owner/‘Mirenyreny’, tied/‘Mifatora’, or fenced/‘Mifefy’). Vaccinations were delivered at no cost to owners, but as animal vaccinations are generally thought of as a paid service in Madagascar, owners were asked how much in Ariary they would be willing to pay to have one animal vaccinated against rabies (after being informed that the current vaccination was free). For each animal vaccinated, we recorded the species (cat or dog), sex, approximate age in years as reported by the owner, and whether the animal had been previously vaccinated.

To assess coverage, post-vaccination coverage surveys were conducted according to a previously established methodology [15,16]. All animals vaccinated were concurrently marked with a colored, nontoxic, livestock crayon along the top of the head or back. At the end of each campaign day between 1600–1800 h, when dogs are most active [15], two transect surveys were conducted on vaccination campaign days in each vaccination location by two teams (consisting of two volunteers and one local guide) for one hour on separate paths and in opposite directions. Marked and unmarked dogs were recorded as well as their roaming status (i.e., roaming, inside a fence, or tied), and their approximate age (>1 year or <1 year of age).

### 2.3. 2019 Campaign

For the 2019 campaign, instead of a static point campaign strategy, vaccine vials (Rabisin) and the supplies needed to administer them (needles, syringes, and vaccination cards for owners) were distributed to the district veterinarian, who then delivered the vaccination at no cost to owners but was directly compensated 1500 Ar (~0.40 USD) per rabies vaccine administered. The campaign lasted from 6 September 2019 to 19 June 2020. One week prior to her visiting each location, the district veterinarian advertised the vaccines by calling ahead to the fokontany leaders and other officials who then advertised to their communities, largely through word-of-mouth. For each animal vaccinated, the district veterinarian collected the animal’s age and sex and asked owners to approximate the distance they travelled to receive the vaccination in meters. Researchers communicated with the district veterinarian about progress periodically throughout the campaign, primarily through telephone calls. No other compensation or instructions were provided, and we asked the district veterinarian to administer as many (or as few) as feasible or wanted. As the vaccinations were delivered continuously, we were unable to do comparable post-vaccination surveys.

### 2.4. Analyses

#### 2.4.1. Campaign Resource and Cost Comparisons

We documented the overall costs and resources required for the two vaccination efforts. We tracked the number of vaccination points, the number of days over which these vaccinations occurred, and the number of person days required overall (i.e., the number of working people per day over the campaign [17]) in addition to monetary costs. As costs were incurred in both USD and Ariary and as the exchange rate declined rapidly between 2018 and 2019, we used the midpoint between the two years (3314 Ar to 1 USD) for the cost comparisons.

For the 2018 campaign, we broke costs down into the following categories: direct vaccine costs (cost for vaccine, syringes, needles, and vaccination cards), supplies (livestock crayons, muzzles, gloves, alcohol, and swabs), food and lodging for NGO personnel and other vaccinators during the campaign, personnel costs (per diems for the district veterinarian, livestock field officers, local guides, and NGO employees), and advertisement (posters and banners for advertising the campaign). Foreign NGO volunteer expenses for travel to Madagascar were not included in these costs. Vehicles and drivers were also not included in these costs, as the drivers’ time and vehicle use were donated to the campaign by volunteers involved in the campaign. In 2019, costs were split into two categories, direct vaccine costs (for the same items as in 2018) and personnel costs (per vaccine fee paid to the district veterinarian), and supplies (a generator and fuel for the veterinarian to maintain the vaccine under cold chain during power outages). Transportation costs were also not included as the district veterinarian used her own vehicle and vaccinated as part of their routine veterinary service provisioning.

We used the data on owners’ reported willingness to pay for vaccines to estimate the proportional reduction in animals vaccinated as fees are increased. We also estimated how this would impact cost per animal vaccinated by approximating the costs for implementation (i.e., those costs that remain fixed) from costs incurred per animal vaccinated (i.e., vaccine, syringe, vaccination card, and per vaccination fee to the district veterinarian in 2019) and by calculating the balance between the returns from owner payments (i.e., increases in cost recovery per animal vaccinated) vs. decreasing numbers of animals vaccinated overall.

#### 2.4.2. Coverage Estimates

For the 2018 campaign, we used the transect data to estimate vaccination coverage as the proportion of dogs sighted that were marked using a binomial confidence interval at the commune level. For the 2019 campaign, we estimated the vaccination coverage using human-to-dog ratios (HDRs) and human population estimates [18,19]. We used a ratio range of 8–25 humans-to-dogs, based on previous data from Madagascar [20] and based on recent estimates from household surveys in the Moramanga District [21]. We set the point estimate using an HDR of 19.5, the midpoint between the HDRs estimated for two communities in the district by LeBlanc et al. 2019. We used human population estimates from the 2018 national census in each commune where the vaccinations were delivered [13]. Coverage was estimated as the number of dogs vaccinated in total in that commune divided by the estimated dog population. We used this same method for the 2018 campaign as well to compare the coverage estimated by the post-vaccination transects vs. HDRs.

#### 2.4.3. Dog Demography

Using the age data on vaccinated animals collected during both vaccination campaigns, we estimated the proportion of population in four age classes: puppies under the age of 1 year, juveniles aged 1–2 years, adults aged 2–6 years, and older dogs aged 6+ years based on broad patterns of survival in comparable dog populations (i.e., low survival in the first year of life, followed by plateauing survival probabilites [22,23]). With the assumption that these estimates represent the population at a stable age distribution, we used a Leslie matrix model to estimate annual adult survival probability and fertility using maximum likelihood estimation [24]. Specifically, we assumed that the number of individuals in each age class follows a Poisson distribution, with the mean predicted by the stable age distribution from the model (the proportion of individuals in each age class at equilibrium, equal to the eigenvector associated with the dominant eigenvalue of the matrix ν) multiplied by the total number of individuals in the population ($N_{t}$):

$$N_{a}∼Pois\left(\nu N_{t}\right)$$

We assumed that all individuals older than 1 year of age reproduce and we did not estimate declines in fertility given the small proportion of dogs older than age six years in the population. To obtain bootstrapped estimates, we used 100 subsampled data sets of 1000 observations each from the observed age data to fit the parameters and varied the initial values used in the optimization (N = 100 initial values sets) for 10,000 parameter estimates total.

#### 2.4.4. Modeling Vaccination Campaign Strategies

We used the parameter estimates from the demographic model to simulate different vaccination strategies in a hypothetical commune with 1000 dogs. We used a discrete time age-structured model with a monthly time step to compare three strategies:

(1) annual vaccination campaigns occurring within the same month each year targeting dogs of all ages
(2) continuous vaccination of new puppies throughout the year targeting puppies that reach the age of 3 months
(3) a combined approach with annual campaigns (as 1) and routine puppy vaccination (as 2)

We split the dog population into puppies (<1 year old) and adults based on the stable age distribution estimated from the demographic model. To estimate pup survival in year one, we took the fertility estimates (i.e., number of new puppies per reproducing dog observed in the pup age class) and divided by an estimate of newborn pups per reproducing dog each year based on average litter size, average number of litters per female per year [7,22,25], and the proportion of the adult population that is female (estimated from our data). We assumed that, for the annual campaign strategy, surviving vaccinated adult dogs were revaccinated in subsequent years [26] but that, if a pup had been vaccinated within 9 months of the campaign, it was not revaccinated. We also assumed that vaccine immunity lasted for a discrete period of 3 years (with revaccination resetting immunity). A subset of parameter estimates resulted in estimates of population decline, but based on the shape of the age pyramid and to simulate reasonable campaign scenarios, we filtered to parameter estimates that corresponded to positive population growth.

### 2.5. Data and Ethics Statement

All data were analysed in R version 4.0.2 (2020-06-22) [27], largely using the tidyverse package suite [28]. Geospatial data were mapped using the sf [29] package. All data and code are archived at https://doi.org/10.5281/zenodo.4663084, accessed on 30 March 2021 and available at https://github.com/mrajeev08/mora_vax, accessed on 30 March 2021. The vaccinations were part of a public health campaign and routine veterinary service provisioning carried out by the local veterinary officials and the NGOs involved and in partnership with the Ministry of Public Health and the Department of Veterinary Services at the national level. MDI also maintained the national research permits (MICET permit: #130-19/MEDD/SG/DGEF/DGRNE) for its research and volunteer programs. Prior to vaccination, verbal informed consent was obtained from animal owners, and owners could opt out of answering any questions or services provided. No personally identifiable information was collected at any point during the campaigns.

## 3. Results

### 3.1. Summary of the 2018 and 2019 Campaigns

During the 2018 campaign, a total of 3137 animals were vaccinated (2057 dogs and 1080 cats) in the Moramanga (urban) and Andasibe (rural) communes over 13 days during the month of April (Table 1). We vaccinated at 7 points in Andasibe and 14 points in Moramanga Ville. During the 2019 campaign, between September 2019–June 2020, the district veterinarian vaccinated a total of 2385 animals (1898 dogs and 486 cats) over 48 days in seven communes in the Moramanga District. While more animals were vaccinated per vaccination point and per vaccination day in 2018 compared to 2019, the number of animals vaccinated per person-day was much higher for the 2019 campaign. More animals were vaccinated in 2018 vs. 2019, but this was largely a result of vaccinating more cats during the 2018 campaign (Table 1).

In 2018, 15% of dogs had a previous history of vaccination (largely in the urban commune of Moramanga Ville), with only 7% of dogs vaccinated within the last year. This remained largely the same in 2019 (~13%), as the district veterinarian focused their efforts in other communes. The district veterinarian did vaccinate 771 dogs in Moramanga Ville in 2019, and of those, 24.9% had been vaccinated in the previous year’s campaign whereas only 4.9% of dogs vaccinated in all other communes had any history of previous vaccination. In addition, in 2019, 2.1% of animals had been spayed or neutered, reflecting efforts by the Mad Dog Initiative to implement free spay and neuter clinics in the district.

In 2018, 19% of owners reported that their animals were free-roaming, but this varied by location (Table 1). In addition, while less than 19% of owners in Moramanga Ville reported that their animals were free roaming, the majority of animals observed during the transects (77%) were observed outside of fences and not tied, and thus, the majority of animals could be classified as semi-confined in the more urban township of Moramanga Ville and free-roaming in the rural setting of Andasibe (approximately 67% of owners reported their dogs as free-roaming, Table 1). In 2019, the district veterinarian also asked owners to approximate how far they travelled in meters to get their animals vaccinated and 94% of people reportedly travelled less than 1 km to reach the vaccination point.

### 3.2. Cost Comparison and Willingness to Pay

**Figure 2:**
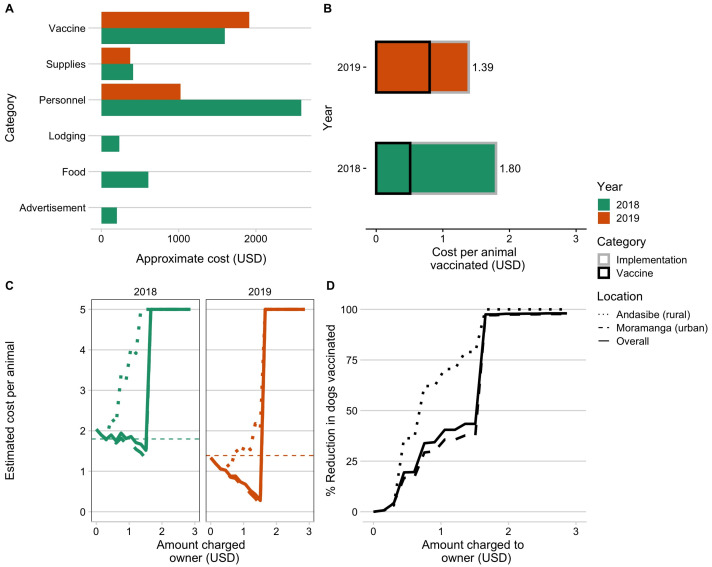
Comparing campaign costs and willingness to pay. (**A**) Vaccine costs broken down by category and by year (colors). (**B**) Overall cost per animal vaccinated for the two campaign years split by direct costs of vaccination per animal (i.e., vaccine, vaccination card, and syringes) and baseline implementation costs (i.e., personnel, supplies, subsistence costs for vaccinators during the campaign). (**C**) Estimated cost per animal vaccinated under a willingness-to-pay model for two campaigns examining increasing costs charged to the owner, with estimated costs declining due to cost recovery through owner payments and then peaking once owners reported no longer being willing to pay for the vaccine. (**D**) The percent reduction in number of animals vaccinated given owners’ willingness to pay. The curves in C and D are shown based on the overall responses to willingness to pay (solid line) from both Moramanga and Andasibe, and the responses split by commune (dashed and dotted lines).

The 2018 campaign cost more overall and per animal vaccinated than the 2019 campaign, largely due to increased personnel costs (Figure 2A,B and Table 1). Reflecting the extra personnel necessary to run the static point campaign, the 2018 campaign also took substantially more person-days per animal vaccinated (Table 1). We found that charging owners for vaccinations would result in minimal cost recovery (Figure 2C) and, beyond a minimal cost, would actually result in increased costs per vaccinated individual than free-of-charge campaigns. Cost recovery would be more likely given a 2019 style strategy, where the majority of the costs are incurred on a per animal basis, compared to the 2018 campaigns, where the costs were largely due to setting up the static point vaccination stations (Figure 2A,B).More importantly, in all cases, even a nominal fee would significantly reduce the numbers of dogs vaccinated and thus vaccination coverage, particularly in the rural commune of Andasibe (Figure 2D).

### 3.3. Comparing Campaign Coverage Estimates

**Figure 3:**
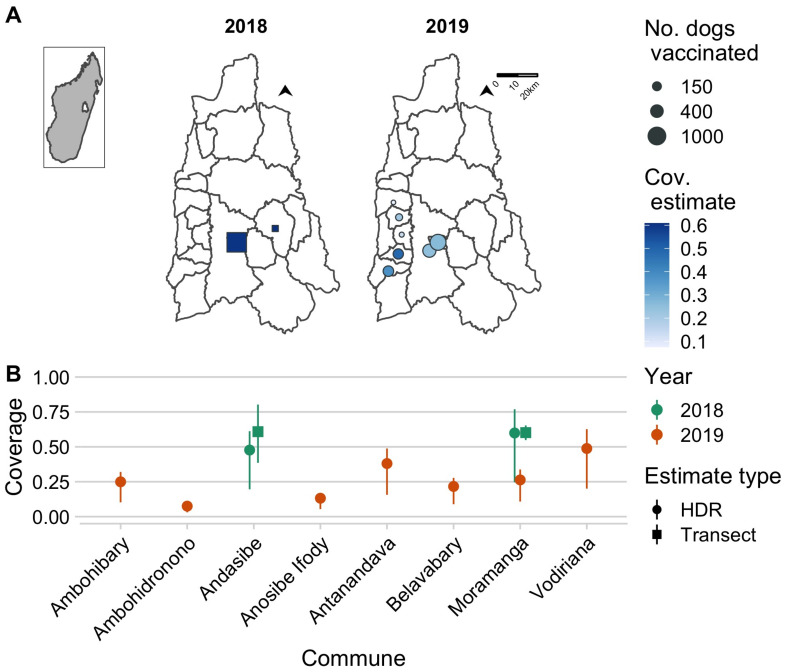
Estimates of coverage achieved by the 2018 and 2019 campaigns. (**A**) The commune-level numbers of dogs vaccinated (size of points) and the associated coverage estimates (color of points) for the year 2018 (squares, estimated using post-vaccination transects) and 2019 (circles, estimated using a human-to-dog ratio (HDR) of 19.5, based on a recent household survey in the Moramanga District). The inset shows the location of the Moramanga District in Madagascar. (**B**) A comparison of coverage estimates by location and by method of estimation (shape of points correspond to post-vaccination transects vs. HDR-based estimates); for transect-based estimates, the line range shows the 95% exact binomial confidence interval, while for the HDR-based estimates, the line range shows the range of coverage estimates assuming an HDR range of 8–25 according to estimates from the literature.

The 2018 campaign covered two communes and was estimated to have achieved approximately 60% coverage (Figure 3A). The 2019 campaign covered seven communes but was estimated to have achieved lower coverage levels (Figure 3, ranging from 5–60%). In the 2018 campaign, we used post-vaccination coverage transects to estimate vaccination coverage, but we were unable to do this in 2019 given the continuous delivery strategy. In addition, in Andasibe in 2018, coverage estimates were based on a single transect resulting in more uncertainty. However, coverage estimates from the transects in 2018 were consistent with the HDR-based estimates (for both Andasibe and Moramanga, transect-based estimates fell within the range of the HDR estimates). We also back-calculated HDRs given our vaccination coverage estimates, and these were similar to the HDRs calculated from the household survey (15.7–32.8 for Andasibe compared to 21.7 in a rural community and 17.8–21.2 for Moramanga Ville compared to 17.2 in an urban community).

### 3.4. Dog Demography and Simulating Vaccination Strategies

**Figure 4:**
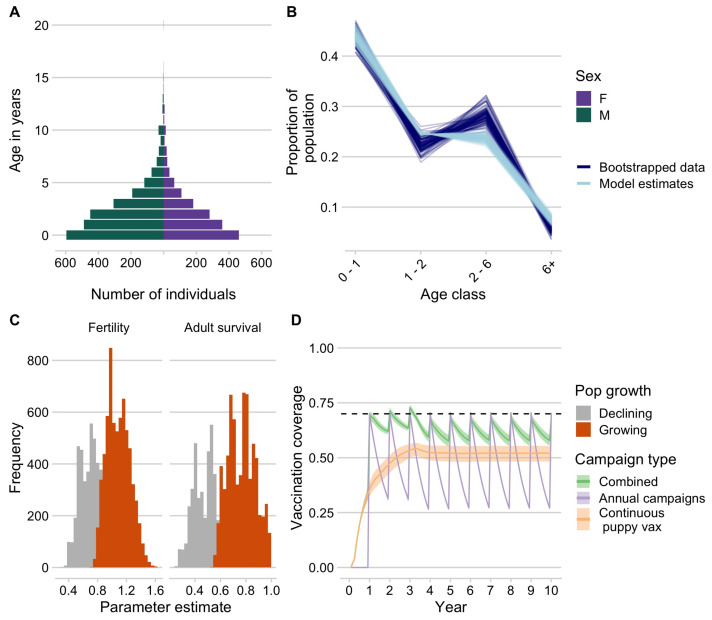
Dog demography and implications for campaign strategies. (**A**) The age pyramid for vaccinated dogs by sex. (**B**) Bootstrapped estimates of the proportion of the population in each age class from the age data (dark blue) compared to estimates from the demographic models fit to these data (light blue). (**C**) Parameter estimates for annual fertility rates and adult survival probability, with estimates highlighted in orange showing the parameter estimates that result in positive population growth. (**D**) Simulated vaccination coverage (N = 1000) using the demographic parameters from (**C**) in a hypothetical commune with 1000 dogs for three different campaign strategies: (1) annual vaccination campaigns targeting dogs of all ages (purple), (2) routine vaccination of puppies at 3 months of age, and (3) a combined strategy with campaigns annually and continuous puppy vaccination in between campaigns.

Demographic data from vaccinated dogs showed a population pyramid with a large base, indicative of a fast-growing population, and with a male bias (approximately 60% male, Figure 4A). We fit these data to an age-structured model and were able to generate parameter estimates, which resulted in stable age distributions consistent with the data (Figure 4B). We filtered parameter estimates that are consistent with a growing population, resulting in mean adult annual survival probability of 0.77 (95% quantile: 0.59–0.97). We used estimates of fertility (on average, 1.09, 95% quantile: 0.82–1.41) to back-calculate pup survival, which ranged from 0.34 to 0.68. We found that, given these demographic parameters, annual campaigns that target dogs of all ages result in rapid decline in vaccination coverage between campaigns, largely due to rapid turnover of the dog population (compared to the impact of waning immunity assuming a discrete 3-year period of vaccine immunity, evident in the additional dip at year 3, Figure 4D). Continuously targeting 70% of the puppy population for vaccination while unable to achieve the peak coverage consistently reached coverage of about 50% of the dog population. A combined strategy maintains the highest and most temporally stable levels of coverage close to the target of 70%.

## 4. Discussion

Through vaccination campaigns implemented in Moramanga District of Madagascar, we saw a high demand for vaccination from dog owners and found that dogs were accessible and able to be handled safely and efficiently for parenteral vaccination at a reasonable cost (between 1.3–1.8 USD per animal vaccinated). We found that providing the vaccine at no direct cost to dog owners will be critical to achieving sufficient coverage, as even with nominal fees, a significant proportion of owners indicated that they would no longer vaccinate their animals. A static point vaccination strategy achieved higher coverage over a shorter time period in 2018 compared to dog vaccinations conducted by the district veterinarian as part of routine veterinary service provision in 2019. However, it came at a higher cost per animal vaccinated, was more limited in geographic scope, and required more resources in terms of personnel. In addition, in the rural setting of Andasibe, the static point campaign strategy achieved lower coverage, reflecting less accessible, hard-to-reach human communities in this location. Based on the lessons learned through these campaigns, in particular, the observation that puppies were relatively easy to handle for vaccinators and owners, we found that continuous vaccination, targeting puppies in particular, may be an effective way to maintain vaccination coverage levels given the high turnover in dog populations.

There were several limitations to our analyses. Owner reports of willingness to pay, age of animals, and distance travelled to the vaccination point likely all suffer from recall bias and uncertainty. Owner-based estimates of age are very coarse, but given the broad age classes we used, they likely are of sufficient precision to capture broad patterns in age structure. However, the ages of the animals brought to vaccination points may not be representative of the age structure of the underlying population. Previous work has shown that, in general, puppies (individuals <1 year) tend to have lower vaccination coverage than adults [30,31,32,33]. Additionally, we did not vaccinate animals less than 1 month of age, which may explain why we sometimes estimated declining populations (as part of the age class of 0–1 years was not captured by the data). Despite these issues, our analyses based on these data are consistent with previous findings from sub-Saharan Africa that demonstrate male-biased populations, skewed towards puppies, and with high mortality in the first year of life [22,34].

Willingness-to-pay studies have been done previously for rabies vaccination and have consistently shown that cost recovery is minimal given the price dog owners are willing to pay for vaccination [35,36,37,38]. In fact, owners generally overstate the amounts they are willing to pay when compared to observed practice [39], and thus, our analysis could underestimate the impact of charging owners on coverage reductions. Similarly, distance to campaign points has been identified as a barrier to vaccination, and in most cases, owners report traveling less than 1 km to reach a vaccination point [32,40]. In the context of Madagascar, these findings are of particular relevance, as animal vaccinations can be mandated by the government but at a cost to animal owners (for example, for the anthrax vaccine in cattle). Importantly, dog owners in the district do believe that vaccination can prevent rabies transmission [21] and, given the observed demand during the campaigns, are amenable to vaccination of their animals. Our results confirm that implementing dog rabies vaccinations as a public health measure and removing as many barriers as possible to dog vaccination will be important to the success of control programs.

We used HDRs, which can be sensitive to underlying estimates of the human population and the spatial scale of estimation [15,41]. However, we used HDR estimates from a recent household survey study in the district and found these to give coverage estimates consistent with those from post-vaccination transects. Finally, in our vaccination model, we made several simplifying assumptions: we assumed that the protective effect of vaccination is lost after three years (simulating vaccination with a long-lasting vaccine such as Nobivac), but we also assumed that vaccinated dogs that survive to the following year are revaccinated in subsequent campaigns (effectively assuming boosting per manufacturer recommendations for Rabisin). Overall loss of vaccine-induced immunity plays a lesser role in declining vaccination coverage given the high population turnover in this context. In our age-structured models, we also do not account for population-carrying capacity, likely resulting in overestimates of growth of the dog population.

One key aspect that we do not consider is potential feedback loops between vaccination and demography. Estimates of the effects of vaccination on dog demography are mixed [22], but vaccination may increase dog survival [42]. If dog population growth is driven by survival, then this could mean that increased vaccination results in increased population growth. However, if growth is driven more by demand from human communities, then vaccination could stabilize the population and reduce population turnover. Improving dog population management, encouraging responsible pet ownership practices, and increasing veterinary services could all complement vaccination efforts but have not been demonstrated to result in meaningful rabies control without parallel dog vaccination [34].

Our estimates of costs per animal vaccinated are in line with recent estimates from other countries [43], although these are likely underestimates given the donation of time and resources by the organizations and individuals involved (i.e., costs associated with international volunteers including airfare and visa costs as well as costs of transportation donated to the campaign). We also did not include costs of pre-exposure prophylaxis as all of our volunteers and vaccinators had been vaccinated prior to the campaigns. Both pre- and post-exposure vaccines should however always be readily available for vaccinators and should be included in vaccination program budgets. While the volunteer-led effort resulted in significant financial and personnel resources being devoted to the campaign, costs were higher overall and per animal vaccinated. In addition, NGO- and volunteer-based campaigns may be difficult to sustain given unpredictable funding, time commitments, and turnover in staff [44]. For volunteer-based efforts, focusing on local volunteers (i.e., veterinary students) may be a more cost-effective strategy. However, similar to international volunteers, volunteers require subsistence during vaccination campaigns when not based in the communities where they study or live. Although we included costs of implementing transect-based coverage estimates, these were negligible (less than 0.10 USD per dog vaccinated), in line with recent studies that have shown that this strategy is a cheap and effective way to estimate coverage [15,16].

Dog vaccination delivered by the district veterinarian was less costly, with the majority of the costs directly related to vaccination. In settings with high dog ownership, moving towards community-based vaccination strategies could be an effective way to achieve sufficient and consistent coverage, particularly in hard-to-reach communities. During our campaign, we found that puppies (aged approximately 1–6 months) were easier to handle compared to adult dogs for both vaccinators and owners (see puppies picutred in basket in Figure 1). Puppy vaccination could be carried out by local officials embedded in communities (along the lines of community health workers who may not have full veterinary qualifications), especially given recent findings on the thermotolerance of rabies vaccines and locally manufactured methods for maintaining temperatures required for sustained vaccine storage (up to 3 months) [45]. Incentivizing vaccinators appropriately will be a key challenge, as currently providing no-cost rabies vaccination is not seen as part of routine duties for district veterinarians or for livestock officers. Implementing dog vaccination alongside government-mandated livestock vaccination campaigns may also be a strategy to scale up vaccination efforts at relatively low cost. Expanding veterinary services across the country and relieving financial pressures on veterinarians and animal health workers through appropriate compensation could greatly improve veterinary services across Madagascar [12].

Overall, our results suggest that dog vaccination is a feasible strategy for controlling canine rabies in Madagascar. However, rabies vaccination must be recognized as a public good. Removing barriers for dog owners and incentivizing veterinarians and other animal health workers to implement vaccination will be key to long-term campaign success. Borrowing strategies from human vaccination efforts, i.e., deploying community health workers, could be a way to deliver vaccinations and to reduce costs in the hardest-to-reach communities. In addition, refining vaccination strategies to local contexts and using improved tools and systems, such as mobile phone-based data collection, could improve efficacy and coverage levels reached [17,46]. To monitor the success of these campaigns, it will be critical to develop efficient and effective methods to estimate vaccination coverage and to measure their impact on reducing rabies incidence through robust surveillance [44,47]. With limited chances for reintroduction from outside the island, implementing community-based mass dog vacciantion campaigns could be a path forward for Madagascar to reach ZeroBy30.

## Figures and Tables

**Table 1:** Summary of the 2018 and 2019 campaigns. Breakdown of the animals vaccinated, prior vaccination history, dog demography, dog ownership, and daily and per vaccination rates by year and location (for 2018).

		2018		2019
	Andasibe	Moramanga Ville	All Communes	All Communes
Total animals vaccinated	528	2609	3137	2385
Total dogs vaccinated	254	1803	2057	1898
Dogs with history of vaccination	5%	16%	15%	13%
Dogs vaccinated within last year	5%	7%	7%	13%
Percent male dogs	55%	56%	56%	65%
Average dogs per owner	0.8	1.1	1.0	–
Percent of owners with free-roaming dogs	67%	19%	28%	–
Animals vaccinated per day (total days)	88 (6)	372.7 (7)	241.3 (13)	49.7 (48)
Animals vaccinated per vaccination point (total points)	75.4 (7)	186.4 (14)	149.4 (21)	37.3 (64)
Animals vaccinated per person day (total person days)	11.7 (45)	21.6 (121)	18.9 (166)	49.7 (48)

## Data Availability

All data and code are archived at https://doi.org/10.5281/zenodo.4663084, accessed on 30 March 2021 and available at https://github.com/mrajeev08/mora_vax, accessed on 30 March 2021.
